# Bernard-Soulier syndrome or idiopathic thrombocytopenic purpura: A case series

**DOI:** 10.22088/cjim.11.1.105

**Published:** 2020

**Authors:** Nahid Reisi

**Affiliations:** 1 Department of Pediatric Hematology and Oncology, Faculty of Medicine, Isfahan University of Medical Sciences, Isfahan, Iran; 2 Child Growth and Development Research Center, Isfahan University of Medical Sciences, Isfahan, Iran; 3 Isfahan Immunodeficiency Research Center, Isfahan University of Medical Sciences, Isfahan, Iran

**Keywords:** Giant platelet, (GP) Ib/IX/V complex, Platelet function disorder, thrombocytopenia

## Abstract

**Background:**

Bernard-Soulier syndrome (BSS) is a rare, autosomal recessive platelet function disorder which is commonly mistaken for idiopathic thrombocytopenic purpura (ITP).The report includes seven cases of BSS that have been diagnosed and treated as ITP for a long time.

**Methods:**

Between 2006 and 2016, data of seven BSS patients who have long been diagnosed and treated as ITP were collected and analyzed.

**Results:**

Two patients were males and 5 were females. The patient's age range was between one day and four years at the onset of symptoms. Easy bruising, nose bleeds and mucocutaneous bleeding were the most frequent symptoms. Bleeding attacks of the gum, gastrointestinal tract and menorrhagia also occurred and in one case bleeding in the injection site of the first vaccination was reported. In 6 patients, parents were relatives and in three cases, there was a family history of low platelet counts. Variable thrombocytopenia, prolonged bleeding time (BT), and large platelets with increased bone marrow megakaryocyte were seen in all cases. Most patients were treated with steroids, Intravenous immunoglobulin (IVIG), and some with IV anti-D, Azathioprine, Danazol, Rituximab. Splenectomy was performed in one case. In supplementary tests the platelet aggregation to ristocetin was absent and GPIb expression level by flow cytometry method was lower than 10%.

**Conclusion:**

BSS should always be considered in differential diagnosis of ITP especially in persistent and refractory ITP.

Bernard-Soulier syndrome also known as Hemorrhagiparous thrombocytic dystrophy is a rare inherited bleeding disorder which affecting the megakaryocyte/platelet cell line, and first described in 1948 by Bernard and Soulier (1, 2). Quantitative or qualitative defect of platelet membrane glycoprotein (GP) Ib/IX/V complex, a receptor for von Willebrand factor (vWF) is the cause of disease (3, 4). It usually inherited in an autosomal recessive manner but there are families with dominant forms (3, 5). The incidence was reported less than 1:1000000 and in countries with high rate of consanguineous marriages it seems to be higher (6, 7, 8). Easy bruising, nosebleeds, gingival bleeding and menorrhagia are common clinical manifestations of the disease and severe life threatening bleeding is rare (3, 6, 9). Symptoms usually begin in early age (1, 8) but can unrecognized until the 3rd- 4^th^decade (3). The severity and frequency of bleeding vary throughout life and diminish with age (1, 9) but menorrhagia and bleeding at the time of childbirth are problems for females (3, 10, 11). Thrombocytopenia, large platelet and prolonged bleeding time are its laboratory findings.

The diagnosis of BSS is usually based on absent response to ristocetin in platelet aggregation studies and low expression of platelet surface GPIb by flow cytometry. Molecular studies can also establish an abnormal genotype (1, 9, 12). Antifibrinolytic agents, desmopressin, platelet transfusion and recombinant factor VIIa are suggested treatments in this disease (13, 14).

This disease due to its clinical and laboratory manifestations has very close similarity with idiopathic thrombocytopenic purpura that is an acquired isolated immune thrombocytopenia. ITP is usually developed by the production of autoantibodies secondary to infections, vaccinations or drugs. Platelet surface receptor antibodies are detectable only in half of patients, and the diagnosis of ITP is one of exclusion. This disease is usually self- limited and observation is enough. Steroids, intraveneous immunoglobulins (IVIG), anti-D globulin, and in chronic cases rituximab, thrombopoietin agonists and splenectomy are treatments (15). Glanzmann thrombasthenia, Von Willebrand disease, May-Hegglin anomaly and gray platelet syndrome are other differential diagnoses of BSS (1, 9). The objective of the present study is a reminder of this rare disease especially in differential diagnosis of unsuccessfully treated or refractory ITP. 

## Methods

In this study were collected clinical and laboratory data of 7 children less than 18 years at Seyed- al - Shohada Hospital in Isfahan, Iran since 2006 to 2016 which were diagnosed and treated as chronic ITP for a several years but due to lack of response to the treatment and clinical suspicion they were re-examined by supplementary tests and the BSS diagnosis is given to them. Demographic and general clinical data including age, sex, time of first bleeding, age of BSS diagnosis, type of bleeding signs and symptoms and family history of low platelet count, abnormal bleeding and consanguineous marriage were collected from patient files. The results of their laboratory findings included platelets count, mean platelet volume, presence of giant platelet in peripheral smear, IVY bleeding time and prothrombin time, activated partial thromboplastin time, level of fibrinogen, vWF antigen and vWF activity, FXIII screening , platelet function tests, bone marrow aspiration and biopsy and flow cytometry were recorded and analyzed.

## Results

Demographic, clinical and laboratory findings and performed treatments in patients are summarized respectively, in table 1, 2 and 3. Two patients were males and 5 were females. The patient's age range was between one day and four years at the onset of symptoms. Easy bruising, nosebleeds and mucocutaneous bleeding were the most frequent symptoms. Bleeding attacks of the gum, gastrointestinal tract and menorrhagia also occurred and in one case bleeding in the injection site of the first vaccination was reported. In six patients, parents were blood relative and in three cases, there was a family history of low platelet counts (table 1). 

**Table1:** Demographic and clinical data in seven BSS patients misdiagnosed as having chronic ITP

**Case7**	**Case 6**	**Case 5**	**Case 4**	**Case 3**	**Case2**	**Case1**	**Variable**
13	1.5	4	7	8	10	17	Age (yr)
female	female	male	female	female	male	female	Gender
4	at birth	2	4	2	3	3.5	Time of first bleeding (yr)
-	-	In cousinry	In brother	-	-	In uncle	Family history of low platelet count± bleeding
+	+	+	+	-	+	+	Consanguineous marriage in parents
13	1.2	3.5	5	7	7	15	Age of BSS diagnosis (yr)
+	+	+	+	+	+	+	Easy bruising
+	-	**-**	+	+	+	+	Epistaxis
-	-	-	-	+	-	-	Gingival bleeding
-	-	-	-	+	-	-	Gastrointestinal bleeding
+	-	-	-	-	-	+	Menorrhagia
-	After first vaccination	-	-	-	-	-	Prolonged bleeding after vaccination, teething or surgery

Variable thrombocytopenia, prolonged bleeding time (BT), and large platelets with increased bone marrow megakaryocyte were present in all cases. In supplementary tests the platelet aggregation to ristocetin was absent and GPIb expression level was lower than 10% of control values (table 2). Most patients were treated with steroids, intravenous immunoglobulin (IVIG), and some with IV anti-D, azathioprine, danazol, rituximab and splenectomy was performed in one case (Table 3).

**Table 2:** Laboratory analysis results in seven BSS patients misdiagnosed as having chronic ITP

**Normal values** **(As per reference interval)**		**Patients values**						**Criteria**
	**Case 7**	**Case 6**	**Case 5**	**Case 4**	**Case 3**	**Case 2**	**Case 1**	
11.5 – 14.5	11	12	11	11.5	10.7	11.6	12	Hemoglobin level (gr/dl)
150 - 450	45-60	61	42	70-104	30-104	43- 90	45- 100	Platelets range (10^3^/mm^3^)
7 - 9	10.3	11.4	15.8	10.5	10.1	12.3	10.5	Mean platelet volume (fl)
few	few	moderate	moderate	few	few	moderate	a lot	Giant platelet in peripheral smear
3 - 7	9.5	15	11	10	13	12	9	IVY bleeding time (min)
10 - 13	13	11	10	11	10	10	13	Prothrombin time (sec)
28 - 38	35	37	33	31	28	28	35	Activated partial thromboplastin time(sec)
30 - 240	Normal	Normal	Normal	Normal	Normal	Normal	Normal	Factor XIII screen (min)
1.5 – 4.5	2.35	1.93	2.46	1.78	2.61	1.78	2.54	Fibrinogen* (gr/dl)
50 – 150%	85	90	78	101	123	88	147	vWF Antigen (Turbidimetric method)
50 – 150%	94	88	95	87	109	96	121	vWF Activity (RiCof method)
50 – 150%	80	108	86	98	148	90	85	F VIII Activity (1-Stage method)
	Normocellular marrow with trilinage hematopoiesis and increased megakaryocyte							Bone marrow aspiration& biopsy
	Absent response to ristocetin, aggregation with ADP, collagen and arachidonic acid							**Platelet aggregation
_	2.2%	2.3%	6.3%	5.1%	1.7%	4.2%	3.4%	Platelet surface GPIb (CD42)

*(Claussmetod), **Platelet aggregation with: Ristocetin (0.75, 1, 1.25, 1.5 mg/ml), ADP (2*10 ^ -5M, 4*10^ -6M, 2*10^ -6M), Collagen (200 micrgm/ml), Arachidonic acid (500 micrgm/ml)

**Table 3:** Performed treatments in seven BSS patients misdiagnosed as having chronic ITP

**Case 7**	**Case 6**	**Case 5**	**Case 4**	**Case 3**	**Case 2**	**Case 1**	**Treatment**
+	-	+	+	+	+	+	Prednisone
+	-	-	-	+	+	+	Intravenous immunoglobulin (IVIG)
-	-	-	-	-	+	-	Anti Rh(D) immunoglobulin (IV anti- D)
-	-	-	-	+	+	+	Azathioprine
-	-	-	-	-	-	+	Danazol
-	-	-	-	+	+	-	Rituximab
-	-	-	-	-	-	+	Splenectomy

## Discussion

In present study we reported seven patients with Bernard-Soulier syndrome which had been treated and followed-up a  long time as ITP, but due to the lack of response to ITP treatments and clinical suspicion, they were re-examined and finally platelet aggregation tests and flow cytometric studies disclose the diagnosis. BSS is a rare, genetically inherited bleeding disorder which is due to its rarity, less consider in patients with thrombocytopenia and often misdiagnosed with ITP (16, 17). There are clinical and laboratory clues that can help differentiate these two: BSS is usually an autosomal recessive disorder, so it is more common in the countries with very high proportion of consanguineous marriages (18). Iran (3, 6), Pakistan and the Arab countries, are areas that are more likely to be affected (5). In report on the annual global survey 2017 of World Federation of Hemophilia that has reported 667 cases of BSS from 113 countries, Iran was at the top with 100 cases (18). Nowadays, the number of reported cases of countries with a lower percentage of consanguineous marriages like United Kingdom, Brazil and France are also high (18). The reason for this may be due to better diagnostic facilities, more accurate records of the disease or the increase in immigration to these countries. 

The other clue which can help in the diagnosis of BSS and other hereditary thrombocytopenia from ITP is the presence of low platelet count in other family members (19, 20). ITP is an acquired disease and usually happens in one of the family. Therefore, the presence of family history of thrombocytopenia in ITP patients is a factor that can question the diagnosis. Accompanying the early onset bleeding at birth, mental retardation, cataracts, hearing loss, absent radius and renal failure with thrombocytopenia are other clues which we must think about hereditary thrombocytopenia in ITP patients (19). In our series, 6 groups of parents were relatives, 3 cases had family history of thrombocytopenia and one case had a bleeding event at birth and these findings helped to diagnose BSS. 

In addition to clinical signs and symptoms, laboratory findings can also helpful in differentiating ITP and BSS. Prolonged bleeding time especially its inconsistency with platelet count (19), the presence of large platelets in peripheral blood smears and increased mean platelet volume (20) are laboratory findings which should be suspected to BSS in thrombocytopenic patients.

 ITP is usually a self- limited disease and majority of patients will improve within 6 months. Furthermore 20-30% of affected children may develop chronic ITP (lasting for more than 12 months). Intravenous immunoglobulin (IVIG), corticosteroids or anti-D immunoglobulin is first line therapy and splenectomy, immunosuppressive therapy or rituximab are in the second line for these patients. In recent years, thrombopoietin (TPO) receptor agonists (romiplostim and eltrombopag) are used in refractory chronic ITP (21). 

Lack of response to these therapies that are usually used for ITP treatment is one of the most important factors which should be suspected to diagnosis (19). In our series, the presence of consanguineous marriages in parents of 6 cases, family history low platelet in 3 cases and early onset bleeding at birth accompanied with laboratory findings and lack of proper response to treatment were clues which led to the diagnosis. In the past, there were also reports of BSS cases that had been misdiagnosed with chronic or refractory ITP and even treated as is for a long time period (16, 19, 22) but despite these reports, these two diseases have always been confused with each other.

In cconclusion based on the very close similarities of BSS with ITP, this disease should always be considered in differential diagnosis of ITP especially in persistent and refractory ITP. 
